# Bioavailability of tocotrienols: evidence in human studies

**DOI:** 10.1186/1743-7075-11-5

**Published:** 2014-01-13

**Authors:** Ju-Yen Fu, Hui-Ling Che, Doryn Meam-Yee Tan, Kim-Tiu Teng

**Affiliations:** 1 Malaysian Palm Oil Board, 6 Persiaran Institusi, Bandar Baru Bangi, 43000 Kajang, Selangor, Malaysia; 2 Department of Molecular Medicine, Faculty of Medicine, University of Malaya, 50603, Kuala Lumpur, Malaysia; 3 International Medical University, No. 126, Jalan 19/ 155B, Bukit Jalil 57000, Kuala Lumpur, Malaysia; 4 Nutrition Unit, Division of Product Development and Advisory Services, Malaysian Palm Oil Board, 6 Persiaran Institusi, Bandar Baru Bangi, 43000, Kajang, Selangor, Malaysia

**Keywords:** Tocotrienols, Bioavailability, Human, Metabolism, Absorption, Vitamin E, Palm oil

## Abstract

As a minor component of vitamin E, tocotrienols were evident in exhibiting biological activities such as neuroprotection, radio-protection, anti-cancer, anti-inflammatory and lipid lowering properties which are not shared by tocopherols. However, available data on the therapeutic window of tocotrienols remains controversial. It is important to understand the absorption and bioavailability mechanisms before conducting in-depth investigations into the therapeutic efficacy of tocotrienols in humans. In this review, we updated current evidence on the bioavailability of tocotrienols from human studies. Available data from five studies suggested that tocotrienols may reach its target destination through an alternative pathway despite its low affinity for α-tocopherol transfer protein. This was evident when studies reported considerable amount of tocotrienols detected in HDL particles and adipose tissues after oral consumption. Besides, plasma concentrations of tocotrienols were shown to be higher when administered with food while self-emulsifying preparation of tocotrienols was shown to enhance the absorption of tocotrienols. Nevertheless, mixed results were observed based on the outcome from 24 clinical studies, focusing on the dosages, study populations and formulations used. This may be due to the variation of compositions and dosages of tocotrienols used, suggesting a need to understand the formulation of tocotrienols in the study design. Essentially, implementation of a control diet such as AHA Step 1 diet may influence the study outcomes, especially in hypercholesterolemic subjects when lipid profile might be modified due to synergistic interaction between tocotrienols and control diet. We also found that the bioavailability of tocotrienols were inconsistent in different target populations, from healthy subjects to smokers and diseased patients. In this review, the effect of dosage, composition and formulation of tocotrienols as well as study populations on the bioavailability of tocotrienols will be discussed.

## Introduction

**Figure 1:**
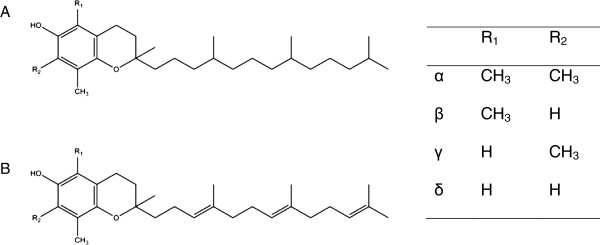
**Chemical structures of tocopherol (A) and tocotrienol (B) with homologues named according to the degree of methylation at R**_**1 **_**and R**_**2**_**.**

The biological role of tocotrienols, as minor components in vitamin E, has been largely underestimated despite studies showing their unique physiological functions. In fact, tocotrienols possess similar structures to tocopherols characterized by a chromanol head named by α, β, γ or δ according to the position and degree of methylation [1,2]. Tocotrienols are differentiated from tocopherols by the degree of saturation at the side chains having 3 double bonds at carbons 3, 7 and 11 whereas tocopherols possess saturated phytyl side chains (Figure 1). Collective studies found that tocotrienols exhibit interesting biological activities not shared by tocopherols, including neuroprotective, radio-protective, anti-cancer, anti-inflammatory and lipid lowering properties [2-8]. Nevertheless, scientific recognition for tocotrienols remains limited compared to tocopherols, being a form of vitamin E widely accepted by the public. One of the reasons is the low abundance of tocotrienols in food sources. In fact, many are not aware that up to 70% of vitamin E from crude palm oil consists of tocotrienols whereas annatto contains the richest source of δ-tocotrienol [9-11]. Red palm olein on the other hand contains up to 541 ppm of tocotrienols together with a bouquet of palm phytonutrients [12]. Another reason that hurdles the advancement of tocotrienols is the lack of understanding on the bioavailability and behavior of tocotrienols in the human system. Although vitamin E in general has been well studied for their absorption and metabolic fate, the plasma concentrations of tocotrienols were found to be much lower compared to tocopherols. When the route of administration was investigated, the absorption of tocotrienols was negligible when administered via the intraperitoneal and intramuscular route, while incomplete absorption was observed when given via the oral route in rats [13]. Being a lipophilic compound, the absorption of vitamin E is essentially dependent on the degree of lipolysis and food intake when administered orally. Since then, several studies have undertaken the initiative to formulate tocotrienols in water soluble delivery systems to increase their solubility. The use of hydrophilic polymers such as cyclodextrin and emulsifiers including Tween 80 showed improved absorption and higher plasma concentrations when administered to rats [14,15]. Intravenous administration via tail vein injections was made possible for tocotrienols by formulating into suitable nano-carriers [16]. When encapsulated in a tumour-targeted vesicle system, tocotrienols are highly promising as a potential therapeutic system to eradicate human epithelial tumours and melanoma tumours in murine xenografts [17].

Although most studies were conducted in rodents and animals, they serve as a basis for clinical evaluations to establish their health benefits in humans. Several reviews have clearly summarized the unique properties of tocotrienols including their antioxidant, anticancer, cardioprotective and neuroprotective effects to name a few [8,18]. In addition, reviews by Kannappan *et al.* and Nesaretnem *et al.* provided extensive insights into the molecular targets of tocotrienols, especially in cancer and inflammation [2,3,19]. Over the past decade, a fair number of human studies for tocotrienols had emerged. Nevertheless, questions on the bioavailability of tocotrienols are complicated by many factors and remained unanswered. Therefore, it is now timely for us to re-examine these human studies in relative to the bioavailability and therapeutic window of tocotrienols in order to strategize the studies forward.

## Postprandial absorption and pharmacokinetics of tocotrienols

Evidence on the metabolism of tocotrienols is relatively limited when compared with α-tocopherol [20]. Only a handful of papers studied the postprandial distribution of tocotrienols thus far, using single dose tocotrienol-rich fractions (TRF) derived from palm oil [21-23]. Fairus *et al.*[21] reported that tocotrienols were transported in triacylglycerol (TAG)-rich fractions containing chylomicrons and VLDL, LDL and HDL cholesterols after administration of TRF at 1011 mg in healthy subjects. In this study, α-, γ- and δ-tocotrienols were detected in both plasma and lipoproteins in appreciable amount though α-tocotrienol emerged as the major homologue detected. Unlike tocopherols which were distributed equally in all the lipoprotein fractions, tocotrienols were mainly detected in the HDL cholesterol at 4 to 8 hours before clearance. This observation suggests that tocotrienols may go through alternative metabolism pathway which warrants further investigation. Alpha-tocotrienol was suggested to be secreted by small HDL particles whilst α-tocopherol is exclusively secreted in chylomicrons. The small HDL particles selectively distributed α-tocotrienol to organs and tissues high in adipose content i.e. epididymal fat, perirenal fat and skin. In contrast, α-tocopherol was reported to be more evenly distributed based on the fact that LDL receptors are available in all tissues [24]. A clinical study reported that higher concentrations of tocotrienols were observed in adipose tissue surrounding benign than malignant breast cancer tumour [25]. The following study conducted by Fairus *et al*. [23] reported the similar trend of response using lower dose of treatments (526 TRF *vs* 537 mg α-tocopherol). The rapid disappearance of tocotrienols in the plasma within 24 hours triggered much debate on the bioavailability of tocotrienols on metabolic effects. This could be partly due to the low affinity of α-tocopherol transport protein (α-TTP) for tocotrienols. The repacking of α-tocopherol in the liver into VLDL cholesterol suggests the longer shelf life and higher concentrations of α-tocopherol in the plasma. However, it was postulated that α-tocotrienol may be absorbed via an α-TTP independent pathway [20]. In a biodistribution study involving surgical patients, total tocotrienols were detected at about 34 nmol/g in adipose tissues after 4 weeks supplementation of 400 mg tocotrienols daily [26]. It is important to note that considerable amount of tocopherols and tocotrienols were also detected in the brain tissues, which were previously shown to have neuroprotective effects in animal studies. Despite studies showing inconsistent tocotrienol levels in human plasma, a recent report found that plasma vitamin E concentration can be part of a non-invasive measurements to facilitate the diagnosis of Alzheimer’s disease [27]. Indeed, using a standardized and relatively sensitive method, plasma vitamin E levels can be explored as a blood biomarker for disease diagnosis or monitoring.

Several postprandial studies were also designed to investigate the pharmacokinetics of tocotrienols when administered orally. In Yap *et al*. [28], evident changes in the pharmacokinetic parameters were observed when TRF was supplemented under fed or fasted state [28]. Comparisons were made between 8 healthy male volunteers given 300 mg mixed tocotrienols consisting approximately 87 mg α-tocotrienol, 166 mg γ-tocotrienol and 43 mg δ-tocotrienol under fasted state or after a high fat meal. The 24 hours area under curve (AUC_0-∞_) of tocotrienols were shown to be increased by at least 2-fold in the fed state, corresponding with a decrease in the volume of distribution (Vd). When tocotrienol homologues were analyzed individually, the maximum plasma concentrations (Cmax) for α-, γ- and δ-tocotrienol reached 1.83, 2.13 and 0.34 μg/mL respectively. The significant increase in tocotrienol bioavailability under fed state was most probably due to the increase of TAG after a high fat meal, followed by bile secretion. In another study where tocotrienols were given at higher doses (296 mg α-tocotrienol, 284 mg γ-tocotrienol and 83 mg δ-tocotrienol), the peak plasma concentrations of γ-tocotrienol did not seem to increase proportionally, i.e. 2.79, 1.55 and 0.44 μg/mL for α-, γ- and δ-tocotrienol [21]. Nevertheless, TRF used in these studies had a different composition in the context of the ratios of homologues. In a follow up study, similar trends were observed [23]. The peak plasma concentrations of tocotrienol homologues were comparable to the doses given, following the order of α > γ > δ. However, dose is not the only factor that affects the plasma concentrations of tocotrienols reflected in the blood circulation. Essentially, solubility of vitamin E in the intestinal lumen is the major determinant for absorption [29]. Emulsification by bile salt and lypolysis by pancreatic enzymes are essential intraluminal processes for lipid formulations [30,31]. In fact, Yap and Yuen [32] described a tocotrienol formulation in the form of colloidal dispersion for enhanced bioavailability. In this study, tocotrienols were formulated into self-emulsifying systems and their pharmacokinetics was studied in fasted healthy volunteers [32]. In the context of Cmax and AUC_0-∞_, tocotrienols administered with self-emulsifying systems were increased by 2 to 4-folds compared to non-emulsified tocotrienols. Although the half-lives of tocotrienols were reported to be approximately 4 to 5-fold lower than that of tocopherols (4 hours vs 20 hours), a dosing schedule of twice daily is sufficient to reach the steady state within 3 days [28,33]. Results from the above studies facilitated our understanding on the pharmacokinetic parameters and biodistribution of tocotrienols, which are fundamental for the design of chronic clinical evaluations. In addition, these studies provided a guideline that acute doses of mixed tocotrienols ranging from 200 mg to 1011 mg were considerably safe for human consumption and no adverse events was reported.

## Clinical evidence and bioavailability

In an effort to determine the therapeutic window for tocotrienols, a number of long term clinical studies were carried out using TRF and tocotrienol derivatives. The majority of these trials were focused on lipid profile as tocotrienols were found to inhibit HMG-CoA reductase [34-36]. However, the optimum dosing regimen to induce therapeutic effects remained unclear. Essentially, the treatment efficacy will depend on an array of variables including: 1) dose, 2) formulation, and 3) study populations. These factors will be discussed as follows.

### Dose

In 1991, Tan and researchers found that TRF was able to decrease total, HDL, LDL cholesterol and TAG levels at a dose of 42 mg/day [37]. Several dose escalation studies have been carried out over the next few years measuring lipid profile as the primary outcome. Wahlqvist *et al*. [38], Rasool *et al*. [39] and Rasool *et al*. [40] did not find significant changes in lipid profile after 2 to 4 months supplementation of 60 mg to 320 mg TRF daily [38-40]. Despite the lack of efficacy on lipid profile, Rasool and co-workers reported significant changes in aortic systolic blood pressure (ASBP) and augmentation index (AI) at dosage above 160 mg/day [39]. When self-emulsifying TRF was used, supplementation at 50 mg, 100 mg and 200 mg per day were able to lower the AI, while doses at 100 mg and 200 mg/day resulted in a significant reduction of carotid femoral pulse wave velocity (PWV) [40]. In fact, supplementation at 100 mg/day demonstrated maximum decrease in AI with a reduction of 8.72% from baseline. Interestingly, Qureshi *et al*. [41] reported significant dose-dependent changes in lipid profile at doses of 25, 50, 100, and 200 mg/day [41]. Marked reduction in total, LDL cholesterols and apolipoprotein B-100 levels coupled with elevated HDL cholesterol and apolipoprotein A1 levels were observed with the lowest dose of 25 mg TRF per day. Unfortunately, current evidence is insufficient to conclude the dose-dependent effect of TRF on lipid profile. While arterial compliance seemed to show consistent improvement with increasing TRF doses, more studies are needed to identify the cause of disparity among these studies.

### Formulation

Although TRF is a common term used to describe a mixture of vitamin E rich in tocotrienols, several variations in composition and formulation of TRF are available in the market (Table 1). Using palm-based TRF (Palmvitee), Qureshi and co-researchers found that daily supplementation of 200 mg TRF significantly reduced total, LDL cholesterols and apolipoprotein B-100 levels while HDL cholesterol, TAG and apolipoprotein A1 levels remained unchanged [42]. Platelet factor 4 (PF_4_) and platelet aggregation were also suppressed after 28 days of supplementation. Contrary to Qureshi’s findings, Mensink *et al*. [43] reported no significant changes on lipid profile and platelet aggregation in hypercholesterolemic subjects when supplemented with TRF preparation [43]. Similar observations were reported by Rasool *et al*. [40] using self-emulsifying TRF preparation (Tocovid Suprabio®) [40]. However, when the dose was increased to 300 mg/day, marked reductions in total and LDL cholesterol levels, as well as total/HDL cholesterol and LDL/HDL cholesterol ratios were observed without changing the HDL cholesterol and TAG levels [44].

**Table 1:** Summary of available tocotrienol formulations including source, delivery systems, excipients and composition of tocorienol homologues

**Tocotrienol formulations**	**Source**	**Dosage form**	**Delivery system**	**Excipients and vehicles**	Composition of tocotrienol homologues (% w/w) *	**Reference**
Palm Vitee	Palm	Capsule	Oil suspension	Palm olein	α: 12-15%, γ: 35-40%, δ: 25-30%	[37,38,42,45-47]
Tocovid Suprabio®	Palm	Capsule	Self-emulsifying drug delivery system	Tween 80, Labrasol, Palm olein/Soybean oil	α: 23.5%, γ: 43.2%, δ: 9.8%	[22,26,28,40,44,48]
Tocomin® 50%	Palm	Emulsion	Self-emulsifying drug delivery system	Tween 80, Labrasol, Soybean Oil	α: 10.7%, γ: 21.6%, δ: 6.4%	[32]
Tri® E	Palm	Capsule	Oil suspension	Palm superolein	74% of mixed homologues**	[49-51]
Tocotrienol-rich fractions (TRF)	Palm	Capsule	Oil suspension	Vegetable oils	α: 34.6%, γ: 24.6%, δ: 15.0%,	[39]
					α: 29.3%, β: 3.0%, γ: 28.1%, δ: 8.2%	[21]
					α: 29..8%, β: 2.9%, γ: 27.0%, δ: 8.6%	[23]
					66.7% of mixed homologues**	[43]
	Rice Bran	Capsule	Oil suspension	Vegetable oils	α: 14.6%, β: 2.2%, γ: 38.8%, δ: 29.9%	[36]
					TRF25 containing α: 15.5%, β: 1.6%, γ: 39.4%, δ: 5.2%, desmethyl and didesmethyl: 20.9%	[41,52,53]
					Various compositionally different tocotrienols supplements	[54]
Individual tocotrienol homologues	Palm	Capsule	Oil suspension	Olive oil	30 mg of γδ-tocotrienols**	[55]
				Medium-chain triglyceride	α, γ and δ-tocotrienyl acetate	[56]

*% w/w of tocotrienol homologues calculated based on g / 100 g of total vitamin E content.

**Composition of individual tocotrienol homologue not specified.

On the other hand, tocotrienols extracted from rice bran oil were found to significantly reduce total, LDL cholesterols, apolipoprotein B-100 and lipoprotein(a) levels, consequently increasing the HDL/total cholesterol and HDL/LDL cholesterol ratios [41,52]. A more prominent decrease in total and LDL cholesterols was observed when the combination of TRF_25_ and lovastatin (HMG-CoA reductase inhibitor) was used [53]. It should be highlighted that subjects participated in these studies were restricted to NCEP Step 1 or AHA Step 1 diet. The implementation of control diet as such may effectively alter the lipid profile, especially in hypercholesterolemic subjects with elevated total and LDL cholesterol levels. The AHA Step 1 guideline restricts daily intake of < 300 mg cholesterol, < 10% energy from dietary saturated fats, and ≤ 30% energy from total fat [56,57]. Adherence to this diet might have an additive or synergistic interaction with TRF on lipid profile, bringing the total and LDL cholesterol levels to an acceptable range [58]. Nevertheless, having 4 homologues within the tocotrienols family, individual homologue might exhibit different potency in their biological effects. Using tocotrienols in the form of acetates, administration of α- and γ-tocotrienyl acetates for 8 weeks (250 mg/day) along with a AHA Step 1 diet did not drastically change the lipid profile although total and LDL cholesterol levels were increased by δ-tocotrienyl acetate [56]. However, a lower rate of the oxidation of LDL cholesterol was observed with the supplementation of α- and δ-tocotrienyl acetates. Similarly, no changes in lipid profile were reported after 8-week supplementation of purified γ- and δ-tocotrienol mixture (120 mg/day) despite the significant reduction in TAG level [55].

As summarized in Table 2, mixed results were observed for changes in lipid markers after long term supplementation of tocotrienols. The inconsistency might be attributed to the different compositions of TRF used. For example, TRF_25_ was extracted from rice bran oil having 17% to 21% of d-desmethyl and d-didesmethyl-tocotrienols which are not present in palm-based TRF [41,52,53]. In addition, the content of α-tocopherol and ratios of the different homologues also vary among the TRF preparations. These variations need to be thoroughly understood before comparative conclusions can be made.

**Table 2:** Summary of changes in lipid profile after chronic supplementation of tocotrienols

**No**	**Reference**	**Study design**	**Subject (F/M)**	**Dose (per day)**	**Duration**	**Finding**
1	Qureshi *et al*. [42]	Double-blind, crossover	25 hypercholesterolemic subjects (11 F/14 M)	200 mg TRF	4 weeks per intervention	Total cholesterol, LDL cholesterol, ApoB: ↓
				300 mg corn oil (placebo)		HDL cholesterol, TAG, ApoA1: ↔
2	Tan *et al*. [37]	Single arm	Preliminary study: 9 healthy subjects (2 F/7 M)	42 mg TRF	30 days	Total cholesterol: ↓ (n = 3), ↔ (n = 6)
		Single arm	Follow-up study: 22 healthy subjects (0 F/22 M)	42 mg TRF	30 days	Total cholesterol:↓ (n =4), ↔ (n = 18)
						LDL cholesterol: ↓ (n = 5), ↔ (n = 17)
						HDL cholesterol: ↓ (n = 3), ↔ (n = 19)
						TAG: ↓ (n = 2), ↔ (n = 20)
3	Wahlqvist *et al*. [38]	Randomized, double-blind, parallel	35 hypercholesterolemic subjects (19 F/ 16 M)	60, 120, 180, 240 mg TRF	16 weeks	Total cholesterol, LDL cholesterol, HDL cholesterol, TAG: ↔
				9, 18, 27, 36 mg TRF in palm superolein (placebo)		
4	Tomeo *et al*. [46]	Randomized, double-blind, parallel	50 subjects with carotid artery atherosclerosis (27 F/ 23 M)	160, 200, 240 mg TRF	18 months	Total cholesterol, LDL cholesterol, HDL cholesterol, TAG: ↔
				300 mg palm superolein (placebo)		
5	Qureshi *et al*. [52]	Randomized, double-blind, parallel	41 hypercholesterolemic subjects (22 F/ 19 M)	200 mg TRF_25_	4 weeks per phase	Total cholesterol, LDL cholesterol, ApoB, Lp(a): ↓
				300 mg tocopherol-stripped corn oil (placebo)		
						HDL cholesterol, TAG, ApoA1: ↔
6	Mensink *et al*. [43]	Randomized, double-blind, parallel	40 mildly hypercholesterolemic subjects (0 F/40 M)	160 mg TRF/d	6 weeks	Total cholesterol, LDL cholesterol, HDL cholesterol, TAG, Lp(a): ↔
				80 mg α-tocopherol (placebo)		
7	O’ Byrne *et al*. [56]	Randomized, parallel	51 hypercholesterolemic subjects (29 F/22 M)	250 mg α-, γ-, δ-tocotrienyl acetate	8 weeks	Total cholesterol, LDL cholesterol: ↑
						HDL cholesterol, TAG, ApoB, LDL/HDL cholesterol ratio: ↔
				250 mg medium-chain triacylglycerols (placebo)		
8	Qureshi *et al*. [53]	Randomized, double-blind, crossover	28 hypercholesterolemic subjects (gender not specified)	50 mg TRF_25_	175 days (35 days per phase)	Total cholesterol, LDL cholesterol, ApoB, Total/HDL cholesterol ratio, LDL/HDL cholesterol ratio: ↓
						HDL cholesterol, TAG: ↔
9	Qureshi *et al*. [41]	Randomized, parallel	90 hypercholesterolemic subjects (15 F/75 M)	25, 50, 100, 200 mg TRF_25_	105 days (35 days per phase)	Total cholesterol, LDL cholesterol, ApoB: ↓
						HDL cholesterol, ApoA1: ↑
				2000 mg tocols-stripped rice bran oil (placebo)		TAG: ↔
10	Baliarsingh *et al*. [36]	Randomized, double-blind, crossover	19 T2DM subjects with hyperlipidemia (9 F/10 M)	6 mg TRF/kg body weight	60 days per treatment	Total cholesterol, LDL cholesterol: ↓
						HDL cholesterol, TAG, Total/HDL cholesterol ratio, LDL/HDL cholesterol ratio: ↔
				100 mg TRF-free rice bran oil (placebo)		
11	Rasool *et al*. [39]	Randomized, double-blind, parallel	36 healthy subjects (0 F/36 M)	80, 160, 320 mg TRF	2 months	Total cholesterol, LDL cholesterol: ↔
				Corn flour (placebo)		
12	Rasool *et al*. [40]	Randomized, double-blind, parallel	36 healthy subjects (0 F/36 M)	50, 100, 200 mg TRF	2 months	Total cholesterol, LDL cholesterol: ↔
				Soybean oil (placebo)		
13	Zaiden *et al*. [55]	Randomized, double-blind, parallel	19 hypercholesterolemic subjects (6 F/13 M)	120 mg γδ-T3	8 weeks	Total cholesterol, LDL cholesterol, HDL cholesterol: ↔
				1200 mg olive oil (placebo)		
						TAG: ↓
14	Yuen *et al*. [44]	Randomized, double-blind, parallel	32 hypercholesterolemic subjects (12 F/20 M)	300 mg TRF	6 months	Total cholesterol, LDL cholesterol, Total/HDL cholesterol ratio, LDL/HDL cholesterol ratio: ↓
				300 mg soya bean oil (placebo)		
						HDL cholesterol, TAG: ↔

↑ = significant increase; ↓ = significant decrease; ↔ = no significant changes, F = female, M = male, T2DM = Type 2 Diabetes Mellitus.

### Study population

A number of clinical trials were conducted to examine the multi-faceted health benefits of tocotrienols in different populations. The bioavailability and efficacy of TRF may vary in different populations. Chin and coworkers [49] compared the absorption of tocotrienols in subjects with different age groups using Tri® E capsules [49]. After 6 months of TRF supplementation, it was observed that plasma tocotrienol levels increased significantly in participants aged above 50 years but not in younger group aged between 35 and 49 years. In accordance with the higher plasma concentrations of tocotrienols, decreased antioxidant enzyme activities, protein carbonyl and advanced glycosylation end products were observed in the elder group. These findings were in agreement with a previous study that reported reduced DNA damage in elderly group aged over 50 years [50]. In contrast, Heng *et al*. [51] reported higher plasma concentrations of tocotrienols in younger individuals (32 ± 2 years old) compared to older participants (52 ± 2 years old) after 6 months supplementation of TRF [51]. Nevertheless in the context of plasma proteome, changes were observed following 3 months of TRF supplementation, in which 6 proteins were up-regulated in the older group whereas only 3 proteins were up-regulated in the younger groups. This finding may suggest higher sensitivity towards TRF supplementation in the older participants, of which lower plasma concentrations were required to induce significant changes in protein expression.

The role of tocotrienols in the modulation of immune system was first established in animal models in 1999 [59-61]. Following that, Radhakrishnan and her team embarked on a clinical trial in healthy subjects to measure changes in T-lymphocytes, B-lymphocytes, natural killer cells and production of cytokines [62]. Although no significant effect was observed after 2 months of TRF supplementation, a further study revealed significant improvement in immune response in subjects challenged with tetanus toxoid (TT) [48]. The participants were supplemented with 400 mg of TRF (Tocovid Suprabio®) per day and received an intramuscular TT vaccination on day 28. Plasma tocotrienol concentrations were increased significantly in the TRF-treated group on day 28 and 56. After 1 month of vaccination, the TRF- supplemented group had a higher increase in anti-TT IgG levels, interferon (IFN)-γ and reduced cytokine secretions compared to the placebo group. Furthermore, Jubri *et al*. [45] reported the effect of TRF supplementation on immune response in cigarette smokers using a daily dose of 200 mg Palmvitee capsules [45]. After 12 weeks supplementation, plasma levels of both tocopherols and tocotrienols were increased although the concentrations of tocopherols were higher. Yet, the plasma levels of tocopherols and tocotrienols showed no difference between smokers and non-smokers. Despite significant improvement in B cells percentage in the non-smoker group, T-cell profile was unaffected by the supplementation of TRF. It was postulated that the amount of TRF supplemented was insufficient to modulate the immune system in cigarette smokers.

In addition, there were several studies investigated the therapeutic efficacy of TRF supplementation on chronic diseases. Tomeo *et al*. [46] investigated the effect of TRF on patients with hyperlipidemia and carotid stenosis [46]. By supplementing the subjects with 300 mg/day of Palmvitee for 12 months, 7 out of 25 subjects with carotid stenosis had improved disease progression. In another study involving patients with Type 2 diabetes mellitus and hyperlipidaemia, the supplementation of 6 mg TRF/kg body weight was found to decrease total lipids, total and LDL cholesterol levels by 25%, 32%, and 45% respectively [36]. The data interpretation may be confounded by the large variations observed at baseline suggesting a better powered study population may be needed. A recent study was conducted in patients undergoing surgical procedures to investigate the biodistribution of tocotrienols [26]. Among the 14 patients awaiting liver transplantation, 50% of patients who had taken TRF supplementation showed a reduction in the model for end-stage liver disease (MELD) score compared to 20% in tocopherol-supplemented patients. In cancer studies, the anti-cancer effect of tocotrienols was first discovered in the early 1990s when Nesaretnam *et al*. [63] reported the anti-proliferative effect of palm tocotrienols in breast cancer cells [63]. Following that, tocotrienols were found efficacious against prostate, pancreatic, colon, gastric and liver carcinomas *in vitro* and *in vivo*[64-68]. The first clinical trial of tocotrienols in cancer patients was embarked in 2006. This trial investigated the synergistic effect of TRF and tamoxifen in women with early breast cancer for 5 years [69]. The pilot clinical trial conducted in Hospital Kuala Lumpur, Malaysia, reported that the risk of dying from breast cancer seems to be reduced in patients taking TRF and tamoxifen compared to tamoxifen alone [3]. Although no significant advantage was observed with the supplementation of TRF in combination with tamoxifen for breast cancer specific survival, the risk of recurrence was 20% less in patients receiving combination treatment. When calculated using the numbers needed to treat, combination treatment may prevent one patient in every 30 from dying due to breast cancer [3]. This study suggested a possible synergistic effect of tocotrienols in combination with tamoxifen in the management of early breast cancer.

Summarizing the above studies, tocotrienols seemed to respond differently to a range of age groups but did not show consistent efficacies in the target study populations. Most of the studies conducted in patients with chronic diseases had relatively small sample size. This demonstrates the need to conduct randomized controlled trials in larger population to confidently evaluate the therapeutic potentials of tocotrienols.

## Limitations/recommendations

Despite the lack of knowledge in the bioavailability of tocotrienols, several reviews in the past have ascertained the physiological functions of tocotrienols derived from both *in vitro* and animal studies collectively [1,8,19,70]. When these effects are not translated in clinical trials, inconsistency in the bioavailability of tocotrienols seems to be one of the major limitations. In fact, outcomes from clinical trials are dependent on various factors at many levels. One limiting factor is the dosage regimen, in which the reference dose for tocotrienols remains uncertain, and is most likely to differ for various indications. In such cases, dosage titration is fairly important in order to determine the optimum dose that demonstrates the highest efficacies and minimal possible side effects. More often than not, plasma tocotrienol concentrations serve as a reference to correlate absorption with therapeutic efficacies. Nevertheless, methods to quantify tocotrienols vary among research groups. A standard monograph for the quantification of tocotrienols is yet to be established, unlike tocopherol derivatives of which their monographs are regularly published in the British, European and United States Pharmacopoeias. Thus, a validated method with sufficient sensitivity for tocotrienols is critically needed. In fact, many analytical and validation studies for tocotrienols are underway, mostly focusing on oil matrix [71,72]. With the establishment of a quantification method, more mechanistic studies can be carried out, including the biodistribution and metabolism of tocotrienols. Debates on the transport pathways of tocotrienols and the role of α-TTP remained inconclusive despite joint efforts from various research teams [1,20]. Imaging studies with tracking system attached to tocotrienols may provide insights into their distribution in tissues and vital organs. In addition, investigations via metabolomics may facilitate our understanding in the context of their metabolic pathways. This technique can provide information on possible interaction, alteration and realistic prediction on pharmacologic response of tocotrienols along their metabolic route. In this review, we identified three major factors affecting the bioavailability of tocotrienols, i.e. dose, formulation and study population. In addition, duration of study and background diet of participants contributed to the research outcomes in most cases. Long term tocotrienols supplementation with dietary intervention may provide accurate insights to the behavior of tocotrienols. Despite being the most convenient form of administration, oral consumption of tocotrienols might not provide the most suitable pharmacokinetic profile for certain indications. Other routes of administration should be explored, such as topical formulations for skin diseases and intravenous formulations for acute treatments. In cancer studies, intravenous formulations can provide a loading dose to achieve an immediate effect, followed by a sustained release formulation to maintain a long term effect. With the increasing knowledge on personalized medicine, formulating tocotrienols into different forms allows them to be used as a versatile therapeutic agent, with the added advantage of high tolerance being extracts from natural products.

## Conclusions

In the context of bioavailability, there are convincing evidence that tocotrienols are detectable at appreciable levels in the plasma after short term and long term supplementations. There is insufficient data on the reference range of plasma concentrations of tocotrienols that are adequate to demonstrate significant physiological effects. Although the pharmacokinetics of tocotrienols are distinctly different from tocopherols which are well studied and remained longer in blood circulation, biodistribution study showed considerable accumulation of tocotrienols in vital organs. In the perspective of therapeutic efficacy, it is evident that the outcome of clinical evaluations is not only affected by the bioavailability of tocotrienols, but also closely dependent on the study designs. In view of the limited understanding, more comprehensive studies in the mechanisms of absorption are warranted.

## Competing interests

The authors declare that they have no competing interests.

## Authors’ contribution

All authors contributed equally to the manuscript. All authors have read and approved the final manuscript.
